# A multicentre evaluation and expert recommendations of use of the newly developed BioFire Joint Infection polymerase chain reaction panel

**DOI:** 10.1007/s10096-022-04538-w

**Published:** 2022-12-07

**Authors:** Kordo Saeed, Nusreen Ahmad-Saeed, Rachel Annett, Gavin Barlow, Lucinda Barrett, Sara E. Boyd, Nicola Boran, Peter Davies, Harriet Hughes, Gwennan Jones, Laura Leach, Maureen Lynch, Deepa Nayar, Robert J. Maloney, Martin Marsh, Olivia Milburn, Shanine Mitchell, Lynn Moffat, Luke S. P. Moore, Michael E. Murphy, Shaan Ashk O’Shea, Fionnuala O’Sullivan, Teresa Peach, Christina Petridou, Niamh Reidy, Mathyruban Selvaratnam, Ben Talbot, Vanessa Taylor, Deborah Wearmouth, Catherine Aldridge

**Affiliations:** 1 Department of Infection, University Hospital Southampton NHS Foundation Trust, Southampton, UK; 2 Clinical and Experimental Sciences, University of Southampton, Southampton, UK; 3 Public Health Wales Department of Microbiology, University Hospital of Wales, Cardiff, Wales UK; 4 Department of Infection, Hull University Teaching Hospitals NHS Trust, Hull, UK; 5 Experimental Medicine & Biomedicine, York Biomedical Research Institute, Hull York Medical School, University of York, Heslington, UK; 6 Oxford University Hospitals (OUH), Oxford, UK; 7 Chelsea and Westminster NHS Foundation Trust, London, UK; 8 Imperial College Healthcare NHS Trust, North West London Pathology, Fulham Palace Road, London, UK; 9 NIHR Health Protection Research Unit in Healthcare Associated Infections & Antimicrobial Resistance, Imperial College London, Du Cane Road, London, UK; 10 Antimicrobial Pharmacodynamics and Therapeutics, Department of Molecular and Clinical Pharmacology, University of Liverpool, Liverpool, L69 3GE UK; 11 Department of Clinical Microbiology, Mater Misericordiae University Hospital, Dublin, Ireland; 12 Department of Microbiology, NHS Greater Glasgow and Clyde, Glasgow Royal Infirmary, New Lister Building, Alexandra Parade, Glasgow, UK; 13 Public Health Wales Department of Microbiology, University Hospital of Wales, Cardiff, Wales UK; 14 Department of Microbiology, Newcastle Upon Tyne Hospitals NHS Foundation Trust, Newcastle Upon Tyne, UK; 15 Department of Orthopaedics, Newcastle Upon Tyne Hospitals NHS Foundation Trust, Newcastle Upon Tyne, UK; 16 College of Medical, Veterinary & Life Sciences, Wolfson Medical School Building, University of Glasgow, Glasgow, UK; 17 Health Protection and Infection Division, Capital Quarter, Public Health Wales, Cardiff, Wales UK; 18 Department of Infection, Hampshire Hospitals NHS Foundation Trust, Winchester, UK

**Keywords:** PCR, Rapid diagnosis, Septic arthritis, Prosthetic joint infections

## Abstract

Septic arthritis is a serious condition with significant morbidity and mortality, routinely diagnosed using culture. The FDA has recently approved the rapid molecular BioFire® Joint Infection Panel (BJIP) for synovial fluid. We aimed to evaluate the BJIP compared to culture and its potential use in patient management. A multicentre retrospective evaluation of BJIP was conducted in the UK and Ireland. Positive percent agreement (PPA) and negative percent agreement (NPA) were calculated between the BJIP and routine culture. A multidisciplinary team (MDT) discussion addressing the optimal or potential case use of the assay practice was facilitated. Three hundred ninety-nine surplus synovial fluid samples (~ 70% from native joints) from eight centres were processed using BJIP in addition to routine culture. An increased yield of positive results was detected using BJIP compared to routine culture (98 vs 83), giving an overall PPA of 91.6% and overall NPA of 93% for the BJIP compared to culture results. The BJIP detected resistant markers and additional organisms that could influence antibiotic choices including *Neisseria gonorrhoeae* and *Kingella kingae*. The MDT agreed that the assay could be used, in addition to standard methods, in adult and children patients with specialist advice use based on local needs. Rapid results from BJIP were assessed as having potential clinical impact on patient management. Organisms not included in the panel may be clinically significant and may limit the value of this test for PJI.

**Supplementary Information:**

The online version contains supplementary material available at 10.1007/s10096-022-04538-w.

## Introduction

Septic arthritis is a serious medical condition with significant morbidity and mortality. Incidences vary between 2 and 10 per 100,000 patients in the Western hemisphere [1–4]. Clinical features of septic (pyogenic or bacterial) arthritis, including periprosthetic joint infections (PJI), can be non-specific or can mimic those of non-septic arthritis (e.g. degenerative, crystal, and other inflammatory arthritis) or aseptic loosening in the case of PJI. The cause of arthritis remains unknown in up to 16–36% of patients [5].

Timely diagnosis of septic arthritis and appropriate treatment are imperative to prevent irreversible joint destruction with consequent long-term disability. Joint aspiration for cytology and bacterial culture, in combination with clinical, radiological, and biochemical findings, remain the standard tests for diagnosis; however, these lack satisfactory accuracy, sensitivity, or specificity. Additionally, while bacteriological culture can be regarded as a ‘‘gold standard’’ diagnostic test, this approach is time-consuming, especially when urgent diagnosis and appropriate antimicrobial treatment are required. Furthermore, negative bacteriological cultures do not always exclude septic arthritis or PJI, particularly if obtained after antimicrobial initiation [6].

Advances in molecular diagnostics have enabled laboratories to use nucleic acid amplification techniques, such as polymerase chain reaction (PCR). This can then accelerate the diagnosis, identify causative agents, and direct targeted antibiotic therapy [6–8]. Depending on the assay (8), PCR can detect a large range of microorganisms, including bacteria/yeast that are fastidious or slow growing, or in circumstances in which previous antimicrobial use may lead to a false-negative culture result. Despite this, multi-plex PCR and/or 16 S rRNA PCR are still not widely available in many diagnostic laboratories routinely [9]. This is mostly due to lack of technical expertise, space, and cost associated with these technologies.

Recently, a rapid and easily applicable BioFire® Joint Infection (JI) Panel (BioFire Diagnostics, Salt Lake City, UT) (BJIP) has been approved by the FDA and CE marking. BJIP is designed to detect 15 Gram-positive (seven anaerobes) bacteria, 14 Gram-negative bacteria (one anaerobe), two yeasts, and eight antimicrobial resistance (AMR) genes from synovial fluid specimens in an hour, allowing for rapid microbiological identification and subsequently targeted adequate antimicrobial therapy. Using synovial fluid culture as a reference standard, it displays a sensitivity of 90.6% and specificity of 99.8% [10]. However, larger scale clinically applicable or real-world data is lacking.

The objective of this study was to evaluate the real sample utility and test performance characteristics of the BioFire JI Panel (BJIP) compared to standard laboratory methods used for the diagnosis of septic arthritis in a multicentre clinical setting and to discuss potential impact assessment, expert recommendations, and future studies required.

## Methodology

### Study type and sites

An observational retrospective comparative analysis was conducted across eight hospital Trusts in the UK and Ireland, including University Hospital Southampton NHS Foundation Trust (Southampton, UK), Chelsea & Westminster NHS Foundation Trust (London, UK), Mater Misericordiae University Hospital (Dublin, Ireland), Oxford University Hospitals NHS Foundation Trust (Oxford, UK), Newcastle upon Tyne Hospitals NHS Foundation Trust (Newcastle, UK), NHS Greater Glasgow and Clyde (Glasgow, Scotland, UK), Hull University Teaching Hospitals NHS Trust (Hull, UK), and Public Health Wales Microbiology, University Hospital of Wales (Cardiff, Wales, UK).

All sites obtained individual approval via their research ethics and/or governance boards and recorded the study a service evaluation project or registered it as a quality improvement project. All data was collected and stored in accordance with the Data Protection Act and the General Data Protection Regulation (GDPR) and anonymised prior to sharing with the wider joint infection study team.

### Study duration and samples

The study lasted 6 months in each of the participating hospitals between March 2021 and March 2022. Surplus (0.5–2 mL) synovial fluid from consecutive joint fluid samples was stored, frozen at − 20 to − 80 °C, unless tested fresh in real time. Routine microbiology diagnostics were performed, including direct and enrichment cultures, according to local standard operating procedures, all based on UK Standards for Microbiology Investigations (SMI) B26 and B44 [11, 12]. Some samples were run in parallel to laboratory workflow based on the availability of staff, but results were not communicated to the treating team.

### Patient selection and sample processing

Where attending clinicians suspected that patients symptoms at presentation were suggestive/suspicious of native or acute PJI with a systemic response, their synovial samples were prioritised for inclusion in the evaluation. The clinical certainty of infection was variable and depended on a number of factors including the suspicion of alternative diagnoses. However, in all cases, the clinical suspicion of infection was high enough to warrant admission and potentially commencement of antimicrobials.

Each centre was allocated 60 investigational use only (IUO) test kits of the BioFire Joint Infection Panel (BJIP) (BioFire Diagnostics, Salt Lake City, UT) as part of this multicentre evaluation program. Synovial fluids (200 µL) were processed according to manufacturer’s instructions using an aseptic technique. Results of the BJIP were not communicated to clinical teams and were not used to influence patient management decisions. Anonymised BJIP and routine results were recorded retrospectively for the evaluation using a Data Collection Tool (supplimentary_1). Results were compared to standard-of-care culture methods, but discordant results were not formally investigated.

### Statistics

Given the nature of the evaluation, descriptive statics were used to compare results from BJIP to the routine culture. Positive percent agreement (PPA) and negative percent agreement (NPA) with 95% confidence interval were calculated based on the results obtained on the synovial fluid cultures for organisms present on the BJIP. The PPA was the proportion of synovial fluid culture positive results in which the BJIP result was positive. The NPA was the proportion of synovial fluid culture negative results in which the BJIP result was negative. Reports on co-detection and resistance markers were noted.

### Expert opinion for impact assessment

Intention for the use of the test and its role in patients’ diagnostic pathways and areas of uncertainty were assessed for each individual centre where medical records were qualitatively reviewed retrospectively by a consultant in microbiology and/or infectious diseases. For each patient, the impact of a positive and negative BJIP result was noted and whether the BJIP results would have potentially influenced patient management in terms of earlier antibiotic treatment, more appropriate treatment, or treatment at all (vs non active treatment) of a pathogen which would otherwise be missed. Additionally, the group (authors) of specialists in the field of microbiology, infectious diseases, diagnostics, and orthopaedic surgery discussed these during a multidisciplinary face to face meeting with representatives from all the participating sites. Recorded discussion and expert opinions were then organised into emerging themes and sub-themes.

## Results

**Fig. 1:**
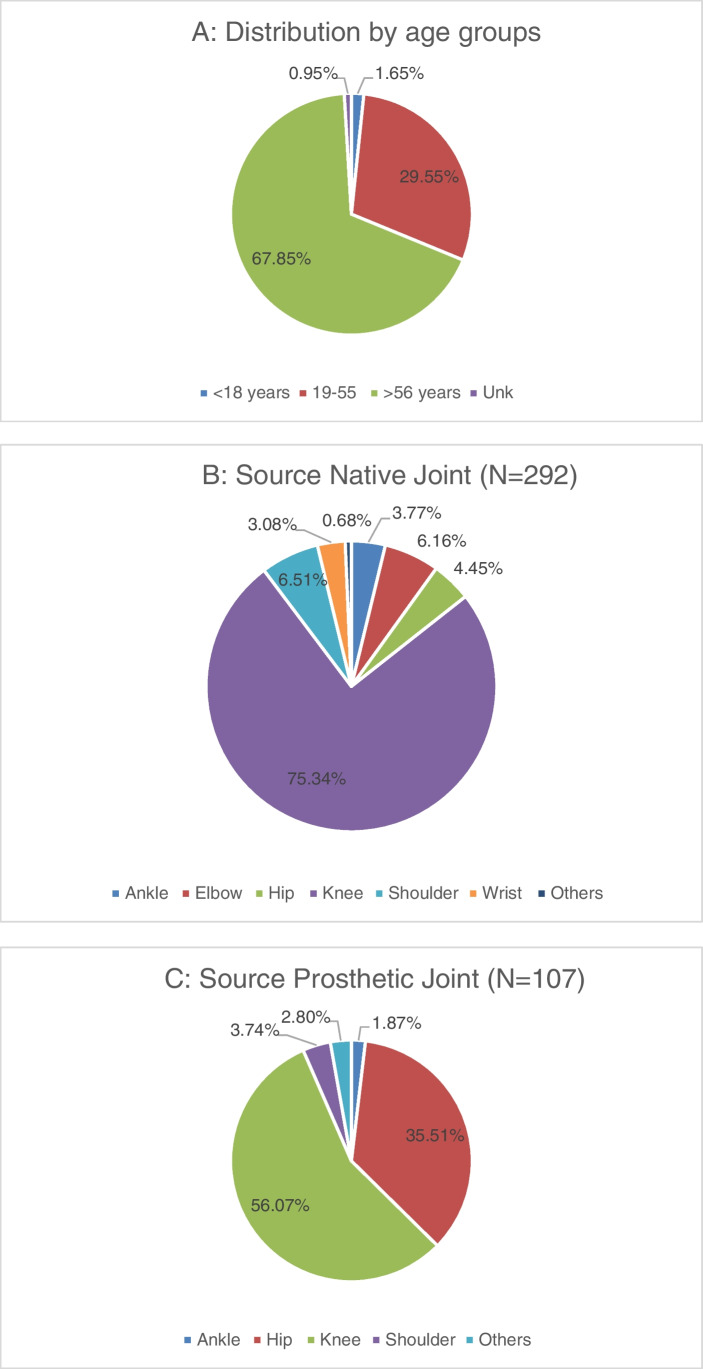
**A**–**C** Distribution of synovial fluid samples by **A** age groups, **B** site location for native joints, and **C** site location for prosthetic joints

During the study period, a total of 399 synovial fluids collected from different sites, sources, and age groups were tested across eight UK and Irish hospitals to evaluate the new BJIP compared to routine microbiology methods (Fig. 1A–C).

The majority of samples (*n* = 294) were negative by both methods. A total of 98 samples had a positive pathogen identification with the BJIP panel compared to 83 samples with routine culture giving an overall positive percent agreement (PPA) of 91.6% with 95% confidence interval [83.6%; 95.9%] and overall negative percent agreement (NPA) of 93% with 95% confidence interval [89.7%; 95.4%] for the BJIP compared to culture results (including enrichment) (Table 1).

**Table 1:** Concordance between BJIP and synovial fluid culture-based methods for native and prosthetic joints. *BJIP* BioFire Joint Infection Panel, *PJI* periprosthetic joint infection

	PJI positive by BJIP	PJI negative by BJIP	Native joint positive by BJIP	Native joint negative by BJIP	Total positive BJIP	Total negative BJIP
Synovial fluid culture positive	36	2	40	5	76 (19.0%)	7 (1.7%)
Synovial fluid culture negative	3	66	19	228	22 (5.5%)	294 (73.7%)

**Fig. 2:**
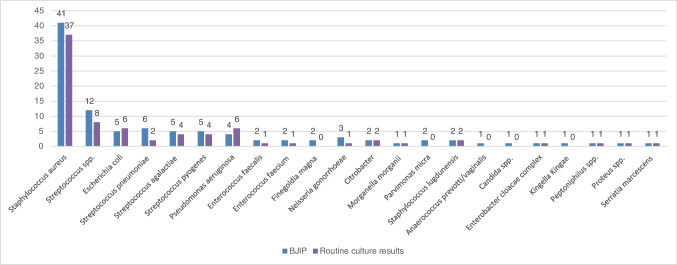
Number of organisms detected by BioFire Joint Infection Panel (BJIP) and routine cultures. Note 24 samples (27 organisms) yield additional positive results by the BJIP

BJIP detected microorganisms in 24 samples (21 native and 3 PJI samples (27 organisms in total)) when the synovial fluid culture was negative (Fig. 2). These organisms included *Staphylococcus aureus*, *Streptococcus* spp., *S. pneumoniae*, S. *agalactiae*, *Enterococcus faecalis*, and *Anaerococcus prevotii/vaginalis*. The BJIP also detected fastidious organisms like *Neisseria gonorrhoeae*, *Kingella Kingae*, *Parvimonas micra*, and *Finegoldia magna* (Fig. 2).

Polymicrobial infection detected by either the BJIP or routine culture was present in 10 samples and is shown in Supplement 2.

Sixteen samples (eight native and eight PJI) grew organisms for which there was no target within the BJIP panel; these include *S. epidermidis*, *S. capitis*, *Cutibacterium* acnes, *Bacillus licheniformis*, *Corynebacterium striatum*, *Dermacoccus nishinomiyaensis*, and *Moraxella osloensis.*

Resistance markers were detected by the BJIP in five samples: 1 CTX-M (ESBL confirmed by culture results), two *mecA* + MREJ (both confirmed as MRSA by routine culture), and there were also two *E. faecium vanA/B* (again one was confirmed, and one was not grown by routine culture). No MRSA, ESBL, or VREs were missed by the BJIP in our cohort.

### Potential clinical use

**Fig. 3:**
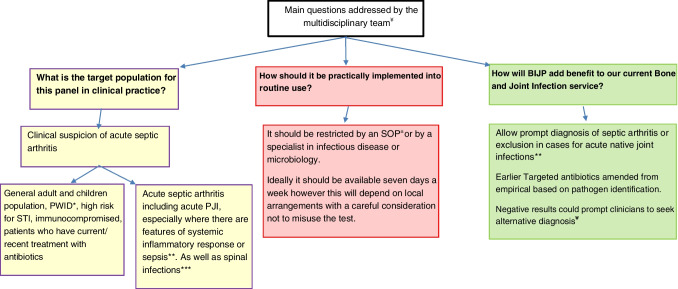
Expert multidisciplinary opinions on potential impact and use of BIJP on the management of septic arthritis in routine clinical practice. ¥ Most of these questions warrants further studies and research in the field; additionally, local experts should be consulted prior to brining the test in to assess how best to incorporate the assay to routine use. ± Standard operating procedure. * Patient who injects drugs. ** For PJI (periprosthetic joint infections) the BJIP does not include *Cutibacterium* and coagulase-negative staphylococci. *** This is off panel; however, the centres who included spinal fluid in the study noted that the BJIP worked very well, in spinal epidural fluids, and in spinal implant fluids. Unlike PJI, spinal implant infections in lumbosacral regions can be polymicrobial, with Gram-negative bacilli, staphylococci, or enterococci representing regional flora on that anatomical area, and a broad range PCR like BJIP would assist in early microbiological identification and subsequent targeted antimicrobial therapy in these cases (please refer to earlier note regarding a limitation of the BJIP which does not include *Cutibacterium* and coagulase negative staphylococci)

A summary of the facilitated multidisciplinary team discussion addressing the optimal or potential case use of this new assay in routine practice condensed to three main themes (Fig. 3).

## Discussion

We report our finding from the evaluation of the new BJIP panel in a real-sample multicentre setting and to consider its potential impact on the management of acute joint infections. The diagnostic yield was higher in the BJIP in comparison with current standard culture methods from synovial fluids, including polymicrobial identification and detection of resistance genes. These can potentially enhance our antibiotic stewardship and targeted treatment in cases of septic arthritis or when seeking alternative diagnosis in the case of rapid negative BJIP.

Delay in diagnosis and inadequate antibiotic therapy in septic joints is associated with worse outcomes, and therefore avoiding this is a priority in the management of acute septic arthritis [13].

The diagnostic performance of the BJIP in comparison to synovial culture demonstrated excellent accuracy, NPA, and PPA in our cohort (Table 1). Early diagnosis of gonococcal septic arthritis, which was detected by the BJIP, is crucial not only for the patients, but also for contact tracing and exclusion of other sexually transmitted infections. In addition, the BJIP was able to detect other organisms that are challenging to grow by conventional culture, for example, it detected the only case of *Kingella kingae* in our cohort which was subsequently confirmed by 16 S -PCR. Another instance where the BJIP successfully identified clinically significant organisms that would have been otherwise undetected by ordinary culture methods included four cases of pneumococcal septic arthritis (Fig. 2). Furthermore, rapid detection of resistant strains like methicillin-resistant *S. aureus (*MRSA), extended spectrum beta-lactamase producers (ESBL), and vancomycin-resistant enterococci (VRE) by the BJIP is more likely to lead to appropriate antimicrobial therapy as well as provision of infection control precautions to prevent onward transmission. This is particularly important in areas with high antimicrobial resistance rates. Additionally, given the range of organisms that the BJIP covers, a negative result, especially in acute native joint infections, would potentially prompt the attending clinician to seek an alternative diagnosis such as crystal or inflammatory arthropathy. However, there were also instances where organisms for which there are molecular targets on the BJIP were not detected by the BJIP happened but isolated on routine culture or enrichment (Table 1), highlighting that this assay should remain complimentary to routine culture methods.

The post hoc multidisciplinary team discussion addressed three main areas of potential impact and use in clinical practice for the BJIP (these centred mainly on the appropriate use of antimicrobials including earlier appropriate antibiotic therapy and rationalisation, shortening of therapy, earlier intravenous to oral switch or outpatient antibiotic therapy (OPAT), and more confidence to avoid or stop antimicrobials, in turn potentially limiting adverse side effects. A negative result on the BJIP could also prompt clinicians to seek an alternative diagnosis or influence the surgical approach, including the potential avoidance of unnecessary surgery. Further studies, ideally randomised controlled trials, will be required to determine the potential clinical impact and cost-effectiveness of the BJIP in real time. Future useful studies should also include the comparison of conventional PCR if available in-house versus BIJP.

Other comments were related to the ease of use, and the reliability for the platform with limited hands-on time from biomedical staff could have an impact on opportunity gains, e.g. staff time saving or concentrating on other diagnostic tests; however, at present the platform is not a substitute for conventional culture, but it will be an adjuvant to what is performed in routine diagnostic laboratories.

The study has several limitations including, but not limited to, different sites performing the study at different time points, although samples were processed using the same principles. The sample inclusion was based on suspicion of infection and availability of surplus samples, exclusions were not recorded, and the number of cases without surplus samples is unknown. Data about prior antibiotic exposure and synovial cell counts is lacking, and discordant samples were not investigated with other PCR methods. Our objective was to compare the test to real-life results as opposed to a pure performance study. Additionally, being a non-interventional study that did not influence antimicrobial prescribing, we can only assume that, based on the opinions of local orthopaedic infection experts, the results of BJIP would have influenced prescribing and antibiotic stewardship. More studies are required to assess the clinical positioning and cost-effectiveness of the panel in future.

The BJIP has limitations in not detecting pathogens in PJI such as coagulase negative staphylococci (apart from *S. lugdunensis*) and *Cutibacterium acnes*. Additionally PJI diagnosis is typically made through the microbiological evaluation of multiple samples rather than just one joint fluid; this may add to consumable and testing costs.

In summary, BJIP had increased yield for on panel organisms compared to routine culture and with rapid turnaround times demonstrates potential clinical impact on patient management. Organisms not included in the panel may be clinically significant, particularly in PJI/ spinal infections, and may limit the value of this test for PJI.

## Supplementary Information

Below is the link to the electronic supplementary material.

Supplementary file1 (XLSX 46 KB)

Supplementary file2 (DOCX 16 KB)

## Data Availability

Available on request.

## Code availability

Available on request.
