# Supplementary material for: A multicentre evaluation and expert recommendations of use of the newly developed BioFire Joint Infection polymerase chain reaction panel
Source: Eur J Clin Microbiol Infect Dis. 2022 Dec 7;42(2):169–76. doi: 10.1007/s10096-022-04538-w (PMC9836977; doi:10.1007/s10096-022-04538-w)
Supplement: Supplementary file 2 — Supplementary file2 (DOCX 16 KB) [file 10096_2022_4538_MOESM2_ESM.docx]

| **Sample Source** | **BIJP results** | **Synovial Fluid culture results** |
| --- | --- | --- |
| Native joint | Streptococcus spp. AND Parvimonas micra | Streptococcus anginosus |
| Prosthetic joint | Enterococcus faecium AND Candida albicans* | Enterococcus faecium AND Candida tropicalis |
| Prosthetic joint | Staphylococcus aureus AND Streptococcus pyogenes | Staphylococcus aureus |
| Prosthetic joint | Enterococcus faecalis AND Citrobacter | Citrobacter |
| Prosthetic joint | Enterococcus faecium AND Citrobacter | Citrobacter |
| Prosthetic joint | Anaerococcus prevotii/vaginalis AND Finegoldia magna |  |
| Native joint | Serratia marcescens | Serratia marcescens AND Staphylococcus epidermidis |
| Native joint | Candida spp. | Coagulase negative staphylococcus AND Candida |
| Prosthetic joint | Enterococcus faecalis AND Citrobacter | Citrobacter AND Enterococcus faecalis |
| Prosthetic joint | Staphylococcus aureus | Staphylococcus aureus AND Finegoldia magna |

**Supplement 1: Polymicrobial detection using BJIP and synovial fluid culture-based methods for Native and Prosthetic Joints**

BJIP: Biofire Joint infection panel
